# Giant Basal Cell Carcinoma of the Scalp with Intracranial Invasion: MRI Findings with Tract Visualisation

**DOI:** 10.1155/2021/6675199

**Published:** 2021-02-10

**Authors:** Maria Brunella Cipullo, Elham Rahimian, Majid Tahsini, Zoi Giavri, Sotirios Bisdas

**Affiliations:** ^1^Department of Advanced Biomedical Sciences, University of Naples “Federico II”, Naples, Italy; ^2^Haghighat Medical Imaging Research Center, Tehran, Iran; ^3^Department of Electrical and Computer Engineering, National Technical University of Athens, Athens, Greece; ^4^Department of Neuroradiology, The National Hospital for Neurology and Neurosurgery, University College London NHS Foundation Trust, London, UK

## Abstract

A rare case of recurrent basal cell carcinoma in the scalp that infiltrated multiple intracranial structures is presented. Basal cell carcinoma represents one of the most frequent malignant nonmelanotic skin neoplasms, but the majority of them have no aggressive and recurrent behaviour. The aim of this case report is to provide an overview of the main clinical and radiologic features of basal cell carcinoma, focusing on the conventional and advanced (tractography) MRI findings and providing an overview of treatment and prognosis.

## 1. Introduction

Basal cell carcinoma is the most frequent lesion of the skin; it is usually localized in sun-exposed parts of the body, so it is often found at the level of the head [1]. The lesion is usually benign but may show an aggressive behaviour with involvement of the underlying bones and distant metastases, highlighting the role of state-of-the-art MRI for its management [2]. Here, we report a case of an aggressive basal cell carcinoma of the scalp with intracranial invasion.

## 2. Case Presentation

**Figure 1:**
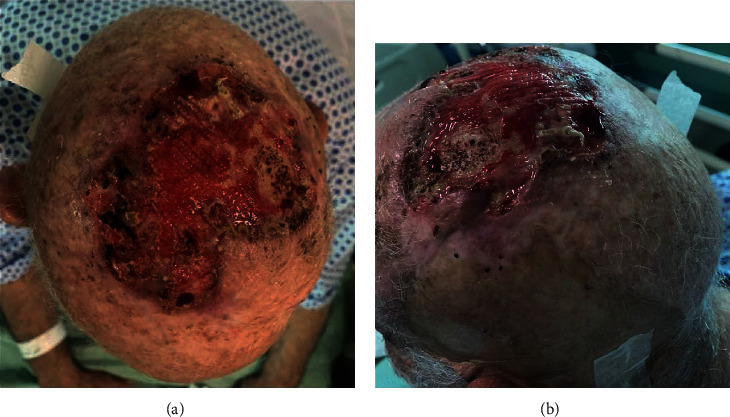
(a, b) Massively ulcerated basal cell carcinoma of the scalp.

We present the case of a 74-year-old otherwise healthy man with a clinical history of an ulcerating skin lesion on the right side of the scalp (Figures 1(a) and 1(b)). The lesion was resected approximately 15 years ago, and biopsy confirmed basal cell carcinoma. Despite multiple treatments in the following years, the patient was referred to our institution with gradual and diffuse enlargement of his cranial vault. He presented with a large ulcer surrounded by erythema on the right side of the scalp. Other symptoms included proptosis of the right globe associated with visual loss and periodical gait disturbance, dizziness, and weakness. A 3 Tesla (T) Magnetic Resonance Imaging (MRI) (Tim Trio; Siemens, Erlangen, Germany) study was performed; the imaging protocol included 3D T1-weighted gradient-echo sequence (MPRAGE) with 176 axial slices (voxel size of 1 mm × 1 mm × 1 mm; repetition time: 1740 ms; echo time: 3.0 ms; flip angle 15; inversion time: 1100 ms) and multidirectional diffusion weighting sequence (repetition time: 3600 milliseconds; echo time: 94 milliseconds; matrix: 128 × 128; slice thickness: 5 mm; *b* = 1000 s/mm^2^; 20 noncollinear diffusion-encoding gradients). All the obtained images were subsequently uploaded and analysed by two experienced neuroradiologists using dedicated cloud-based software (Brainance MD, Advantis Medical Imaging, Eindhoven, The Netherlands).

### 2.1. Imaging Findings

**Figure 2:**
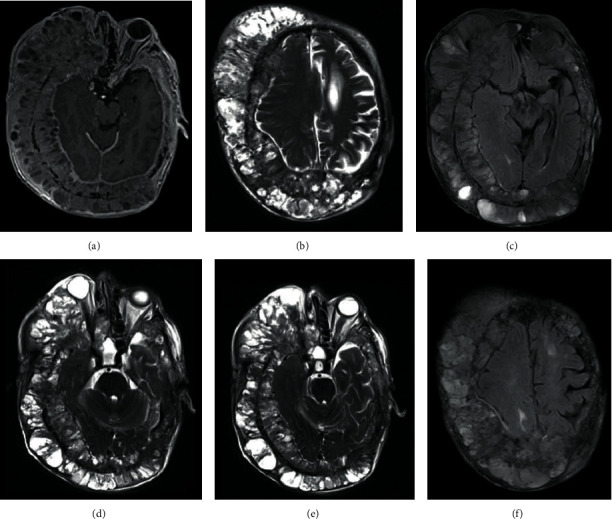
3.0 T MRI with and without administration of paramagnetic contrast agent. On axial postgadolinium T1w-FFE image, there is evidence of (a) an extensive enhancing lesion of the scalp showing, in axial T2-TSE images, infiltration of (b) skin, bones, dura mater, and subdural spaces at the level of the right middle and inferior frontal gyrus. Compression of the right orbital and straight gyrus is also noted, as seen on axial FLAIR-T2w images (c). Axial T2-TSE images show also that (d) the right orbit is fully invaded by the lesion, with proptosis of the globe, which appears compressed, and compression of the optic nerve and that (e) there is invasion of ethmoidal cells, pterygopalatine fossa, and foramen rotundum. Axial FLAIR-T2w images show elevated signal intensity in some white matter fibers, like the right frontal lobe (f).

The MRI showed an avidly enhancing tumour within the cranial vault, extensively involving the right scalp but with extension on the left side, at the level of the occipital pole and vertex (Figure 2(a)). The lesion showed diffuse infiltration of the skin, bones, and dura mater with involvement of the right frontal sinus (Figure 2(b)). Infiltration of the subdural space was noted at the level of the right middle and inferior frontal gyri, with no overt evidence of infiltration of the brain parenchyma (Figure 2(b)). There was also compression of the right orbital and straight gyri (Figure 2(c)). On this side, the orbit was obliterated by the lesion, with proptosis and compression of the globe and optic nerve, which appeared stretched and thinned in comparison to the contralateral corresponding structure (Figure 2(d)). There was also infiltration of the posterior half of the right ethmoidal cells and the right maxillary and sphenoid sinuses extending into the foramen rotundum and pterygopalatine fossa (Figure 2(e)). Temporal bones and mastoid processes on both sides were obliterated with involvement of the carotid canals. The right middle cerebral artery (MCA) appeared slightly laterally displaced by the space-occupying effect of the displaced brain parenchyma as the lesion caused compression of the right cerebral hemisphere convexity. This had resulted in left midline shift of approximately 1 cm and compression of the right lateral ventricle.

In the FLAIR-T2-weighted and T2-weighted images, there was elevated signal intensity in the periventricular white matter of the left centrum semiovale and corona radiata, in the subcortical white matter of the left insula and, in lesser extent, in the juxtacortical white matter of the posterior parietal gyrus and the periventricular white matter of the right frontal lobe (Figure 2(f)).

**Figure 3:**
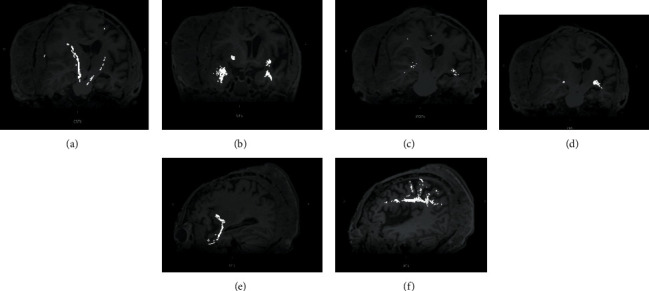
High-resolution tractography shows significant leftward deviation of the right white matter tracts, mainly of (a) corticospinal tract and (b) uncinate fasciculus, with rarefaction of fibers of the (c) right inferior fronto-occipital fasciculus and (d) optic radiation. On a sagittal plane, the (e) left optic radiation and (f) arcuate fasciculus appear fully preserved.

There was no evidence of disruption of the major tracts in the cerebral and cerebellar hemispheres, as demonstrated by the tractography in the diffusion tensor imaging (DTI). However, the intact white matter tracts appeared leftwards deviated with some rarefaction of the right hemisphere (Figures 3(a)–3(f)). No significant differences were found between the respective tracts in two hemispheres in side comparisons.

### 2.2. Follow-Up/Treatment

Given the recurrence of the tumour despite various treatments, including curettage, electrodessication, radiotherapy, and surgical excision, and the problematic patient surveillance, due to his scarce compliance to follow-up appointments, removal of the lesion appears currently very difficult and is deemed to be of high risk. Recently, the patient presented with cellulitis and sepsis. Nevertheless, a regular clinical and radiological follow-up plan has been in place.

## 3. Discussion

Basal cell carcinoma (BCC) is the most frequent malignant skin lesion encountered in the scalp in fair-skinned people, and along with squamous cell carcinoma, they constitute the group of nonmelanoma skin cancers (NMSC). It arises from the basal layer of the epidermis, specifically from stem cells that lie in the interfollicular epidermis of the dermal-epidermal junction and in the bulge of the hair follicle. Due to its origin, Ackerman and Gottlieb proposed a new definition of BCC, describing it as trichoblastic carcinoma [3]. BCC is usually characterized by an indolent behaviour, making surgical excision curative in most cases. It is demonstrated that almost 90% of BCCs occur in the head and neck and be locally aggressive infiltrating adjacent tissues, like bones and muscles. BCCs are characterized by a high rate of skin recurrence but very low incidence (approximately 0.003–0.55%) of nodal metastases [1]. The main sites of metastatic diffusion are locoregional lymph nodes, lungs, and bones. It could rarely metastasize in deeper structures causing perineural spread.

BCC usually affects elderly patients (men more than women), and recognized risk factors are sun exposure, use of photosensitizing drugs, immunosuppression, and ionizing radiation. Its incidence rate is significantly increased in some inherited syndromes, like Gorlin-Goltz syndrome or xeroderma pigmentosum [1]. BCC of the scalp usually appears as an ulcerative, nonhealing skin lesion [4] and rarely as an erythematous or skin nodule [5]. It may be subdivided into two main different groups based on clinicopathologic features, namely, the nodular or superficial BCC and the less frequent variants sclerodermiform/morpheaform, infiltrative BCC, micronodular and fibroepithelioma of Pinkus [1]. CT and MRI are mainly used for preoperative staging of extensive lesions, which are suspicious of deep invasion or metastases. On MRI, BCC is usually hyperintense to muscle on T2-weighted images, iso- or hyperintense on T1-weighted images, and avidly enhancing after gadolinium administration.

There are no clear guidelines on the preferred modality for the evaluation of bone involvement but MRI appears not inferior to CT and possibly more accurate for detecting orbital bone involvement [1]. It has been demonstrated that 1.5 T MRI allows the identification of many histologic patterns of inflammatory skin diseases, but it has some limits in spatial and contrast resolution, while 3 T MRI offers better signal-to-noise ratio (SNR) with the trade-off of more frequent susceptibility and chemical shift artefacts. High-resolution 1.5 T MRI performed with a microscopy surface coil for facial BCC has shown higher accuracy in identifying facial tumour extension and bony infiltration with less artefacts [2]. MRI is also the modality of choice for characterizing perineural invasion though it is rare in BCC. Nevertheless, CT is recommended in the case of suspected body metastases, particularly for the evaluation of potential osseous tissue involvement, and to evaluate treatment response. Regarding scalp and face BCC, CT is also useful for studying maxillary bone involvement complementary to MRI [1].

Giant BCC is a rare variant defined as a tumour larger than 5 cm in diameter [5], characterized by a more aggressive behaviour, with a high rate of deep infiltration of extradermal structures, like bones and cartilage. These features make giant BCCs difficult to remove, and radical surgical resection can result in complex tissue reconstruction [4]. The most appropriate treatment appears to be en bloc resection of all involved tissues, with removal of the dural layer, if involved, too. The main issue in this approach consists in the exposure of neural structures and the risk of reexposure if reconstruction fails. Therefore, it is suggested a single-stage approach in which all separated tissues are reconstructed. In such case, dura is reconstructed using alloplastic materials; cranioplasty is performed using either titanium mesh (which carries the risk of cerebrospinal fluid (CSF) leak) or methyl-methacrylate, whereas for the external surface a free tissue (usually bulky latissimus muscle) is used. Recipient vessels are usually the temporal artery and veins being preferred both for the distance of the lesion from the neck and closeness of the superficial temporal vessels to scalp lesions [6]. In addition to conventional anatomic MRI sequences, for first time in literature, we have applied DTI to analyse the impact of the circumferential aggressive tumour on the underlying intracranial structures. There was evidence of stretching and deviation of the right CST, OR, and IFOF tracts, mainly on the right hemisphere. It is known that white matter tracts which are dislocated by tumour usually remain identifiable in their new location or orientation on DTI maps without changes in the fractional anisotropy values, while oedematous or tumour-infiltrated tracts would show decreased anisotropy [7].

Finally, there are a few case reports [4, 6, 8] showing BCC with intracranial invasion but, to our knowledge, no case report has previously demonstrated such widespread tumour with resulting compressive effect on the cerebral parenchyma.

## Data Availability

Data supporting the information in this case report are in the patient files which are the property of the hospital where he was seen.
